# Plasma troponin T reflects lower motor neuron involvement on electromyography in amyotrophic lateral sclerosis

**DOI:** 10.1093/braincomms/fcaf177

**Published:** 2025-05-06

**Authors:** Sanharib Chamoun, Sofia Imrell, Zane Upate, Ulf Kläppe, Linn Öijerstedt, Solmaz Yazdani, Mikael Andersson Franko, Juliette Foucher, Louisa Azizi, Anikó Lovik, Kristin Samuelsson, Rayomand Press, Fang Fang, Emma Svennberg, Alexander Juto, Caroline Ingre

**Affiliations:** Department of Neurology, Karolinska University Hospital, Stockholm, Sweden; Department of Clinical Neuroscience, Karolinska Institutet, Stockholm, Sweden; Department of Neurology, Faculty of Medicine and Health, School of Medical Sciences, Örebro University, Örebro, Sweden; Department of Neurophysiology, Karolinska University Hospital, Stockholm, Sweden; Department of Neurology, Karolinska University Hospital, Stockholm, Sweden; Department of Clinical Neuroscience, Karolinska Institutet, Stockholm, Sweden; Department of Neurology, Karolinska University Hospital, Stockholm, Sweden; Department of Clinical Neuroscience, Karolinska Institutet, Stockholm, Sweden; Department of Clinical Neuroscience, Karolinska Institutet, Stockholm, Sweden; Department of Clinical Science and Education, Södersjukhuset, Karolinska Institutet, Stockholm, Sweden; Department of Neurology, Karolinska University Hospital, Stockholm, Sweden; Department of Clinical Neuroscience, Karolinska Institutet, Stockholm, Sweden; Department of Clinical Neuroscience, Karolinska Institutet, Stockholm, Sweden; Unit of Integrative Epidemiology, Institute of Environmental Medicine, Karolinska Institutet, Stockholm, Sweden; Department of Neurology, Karolinska University Hospital, Stockholm, Sweden; Department of Clinical Neuroscience, Karolinska Institutet, Stockholm, Sweden; Department of Neurology, Karolinska University Hospital, Stockholm, Sweden; Department of Clinical Neuroscience, Karolinska Institutet, Stockholm, Sweden; Unit of Integrative Epidemiology, Institute of Environmental Medicine, Karolinska Institutet, Stockholm, Sweden; Department of Cardiology, Karolinska University Hospital, Stockholm, Sweden; Department of Medicine, Huddinge, Karolinska Institutet, Stockholm, Sweden; Department of Neurology, Karolinska University Hospital, Stockholm, Sweden; Department of Clinical Neuroscience, Karolinska Institutet, Stockholm, Sweden; Department of Neurology, Karolinska University Hospital, Stockholm, Sweden; Department of Clinical Neuroscience, Karolinska Institutet, Stockholm, Sweden

**Keywords:** troponin T, electromyography, amyotrophic lateral sclerosis, motor neuron disease, biomarkers

## Abstract

Cardiac troponin T (cTnT) is elevated in neuromuscular conditions without apparent cardiac disease, including Amyotrophic Lateral Sclerosis (ALS). The reason for this increase is unclear. Since cTnT is found in both cardiomyocytes and skeletal muscle cells, we aimed to investigate the latter as a possible cTnT source. We examined the correlation of cTnT in venous blood to lower motor neuron (LMN) involvement on Electromyography (EMG). A positive correlation between EMG findings and cTnT levels would indicate that cTnT is a biomarker for LMN involvement in ALS. This observational cohort study was conducted on a tertiary referral centre for neuromuscular diseases in Stockholm, Sweden. Consecutive patients with ALS were included. EMG was performed during diagnostic work-up, and high-sensitive cardiac troponin T (hs-cTnT), plasma creatine kinase (CK), and serum neurofilament light (NfL) were analysed within 6 months of the EMG. King's stage and score on the Revised Amyotrophic Lateral Sclerosis Functional Rating Scale (ALSFRS-R) closest to hs-cTnT sampling were noted. In total, 50 ALS patients diagnosed between 1 January 2014 and 31 December 2018 were included and followed until death, invasive ventilation, or the 14 August 2024. Hs-cTnT correlated positively with the number of muscular regions involved (*τ* = 0.283, *P* = 0.009) and percentage of muscles involved on EMG (ρ = 0.367, *P* = 0.009). Hs-cTnT was associated with the percentage of muscles involved in EMG in the adjusted linear regression. Patients with higher hs-cTnT had more advanced King's stage, both when numerical hs-cTnT and subgrouping high (≥15 nanogram/L) versus normal hs-cTnT was used (τ = 0.253, *P* = 0.021 and *U* = 197.5, *P* = 0.022, respectively). Hs-cTnT was neither correlated to ALSFRS-R total score (ρ = −0.176, *P* = 0.220 and U = 249.5, *P* = 0.233, respectively) nor ALSFRS-R excluding respiratory domain score (*ρ* = −0.069, *P* = 0.632 and *U* = 280.5, *P* = 0.558, respectively). High versus normal hs-cTnT did not predict survival (univariate analysis, HR = 1.824, *P* = 0.060). Numerical hs-cTnT was associated with shorter survival (univariate analysis, HR = 1.635, *P* = 0.017) but not after adjusting for age at diagnosis (HR = 1.413, *P* = 0.105). This study illustrates that ALS patients with higher hs-cTnT have more spread disease as evidenced by the positive correlation between hs-cTnT and both EMG and King's stage. This is not true for established biomarkers of muscle damage (CK) and neuroaxonal damage (NfL). These findings need to be confirmed in larger studies but suggest that hs-cTnT is a biomarker of LMN involvement in patients with ALS and could be used in clinical trials.

## Teaser

Chamoun *et al*. report that cardiac troponin T in plasma is positively correlated with lower motor neuron involvement on EMG in patients with amyotrophic lateral sclerosis. They conclude that this is a possible novel biomarker reflecting the degree of denervation and spreading, to be used in future clinical trials.

## Graphical Abstract

**Graphical Abstract:**
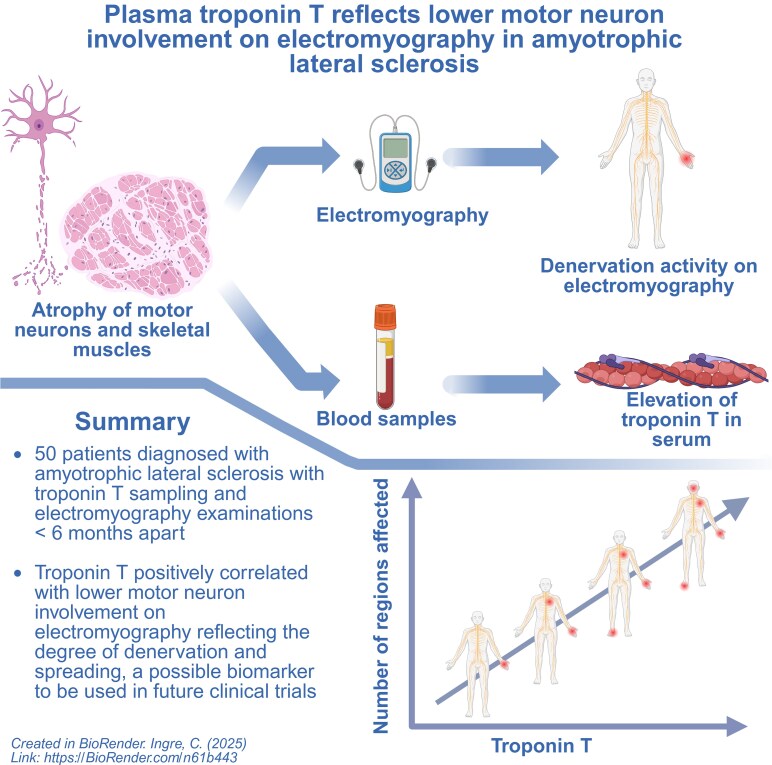

## Introduction

Amyotrophic lateral sclerosis (ALS) is a motor neuron disease characterised by progressive and simultaneous damage to the upper and lower motor neurons (LMNs), leading to neuron degeneration.^1,2^ Clinically at onset, this usually presents as a progressive palsy, most common distal and asymmetrical, with a focal start in either an extremity or as altered speech due to the affected bulbar region.^3^ During the disease course, the respiratory muscles are also affected, ultimately causing respiratory failure and death. The clinical presentation involves LMN symptoms such as cramps, fasciculations, and muscular atrophy, as well as upper motor neuron symptoms of stiffness, spasticity, and weakness.^1,2^ The spreading pattern can vary, and the heterogeneity of the disease makes it difficult to diagnose, commonly delaying the time to diagnosis.^4^ At present, the time to diagnosis is 1 year in most European countries, including Sweden.^5^ The diagnosis of ALS is based on the patient's medical history, neurological examination, neurophysiological studies, magnetic resonance imaging of the brain and spinal cord, as well as analysis of blood and cerebrospinal fluid.

The most widely used biomarker for ALS in clinical practice is the neurofilament light protein (NfL). NfL is a protein found in the cytoplasm of neurons, functioning as a structural part of the cytoskeleton and is hence a biomarker of neuroaxonal damage for both upper and LMNs.^6^ Higher levels correlate with faster disease progression and shorter survival, but do not represent the denervation activity of muscles involved, making it a good biomarker for clinical trials over a longer time, but a weak biomarker for the instant effect of therapeutic impact on the neuromuscular junction.^7,8^ In contrast, creatine kinase (CK) is involved in muscle energy metabolism and leaks into blood upon denervation and atrophy in ALS patients, therefore exclusively reflecting LMN damage.^9^

Neurophysiological measurements reflect the pathogenesis of ALS, provide information for diagnosis, and the disease course and its distribution.^10,11^ For this, various neurophysiological methods are used, including nerve conduction studies, reflex measurements (reflex studies), electromyography (EMG), transcranial magnetic stimulation, excitability studies, neuromuscular ultrasound, motor unit number estimation, motor unit number index, as well as various mathematically calculated indices.^12-18^ A more widespread disease in neurophysiological examinations predicts faster disease progression.^19,20^ The most informative examination is EMG, and parameters and findings from EMG are used to define electrophysiological criteria for ALS.^21^ Conventional EMG studies can reveal signs of active and chronic denervation, both of which are required for the diagnosis of ALS.^2^ Signs of active denervation are fibrillation potentials and positive sharp-waves, whereas large motor unit potentials (MUPs) and reduced interference patterns are signs of chronic denervation.^2^

Troponin T elevation is linked to denervation and the following reinnervation process.^22-24^ The Troponins are a bundle of proteins found in muscle tissues involved in the contraction mechanism of muscle cells. Cardiac Troponin T (cTnT) is a subunit of this protein complex and is mainly known for its usage in diagnosing myocardial damage.^25^ In addition, cTnT might also be elevated in non-cardiac conditions, such as neuromuscular diseases.^26-28^ Our group has previously identified elevated levels of cTnT in blood among Swedish patients with ALS and demonstrated a longitudinal increase, regardless of age, sex, or place of onset.^7^ The longitudinal increase of cTnT in ALS patients has also been described by Castro-Gomez *et al*., who additionally found a negative correlation between cTnT and total Revised Amyotrophic Lateral Sclerosis Functional Rating Scale (ALSFRS-R) score.^29^

The non-cardiac origin of hs-cTnT in ALS has never been investigated. By examining the correlation of hs-cTnT levels measured in venous blood to LMN involvement by using needle EMG findings, additional data for such a link could be provided.

This might suggest that Hs-cTnT could then be used as a biomarker of LMN involvement in ALS and maybe also in other neuromuscular diseases, and could then be used as a screening tool even outside neurological practice, given that it is inexpensive and readily available. Under these circumstances, an abnormal hs-cTnT could put the patient on a ‘fast track’ to a neurophysiologic investigation or to a neurologist, thereby shortening time to diagnosis and proper care. Hs-cTnT could also be used in ALS clinical trials as a biomarker of decreased LMN affection.

The aim of this study was to investigate whether cTnT levels correspond to the disease burden of the LMNs, measured by an extensive needle EMG examination, and to evaluate cTnT levels in relation to survival.

## Materials and methods

### Study participants

**Figure 1:**
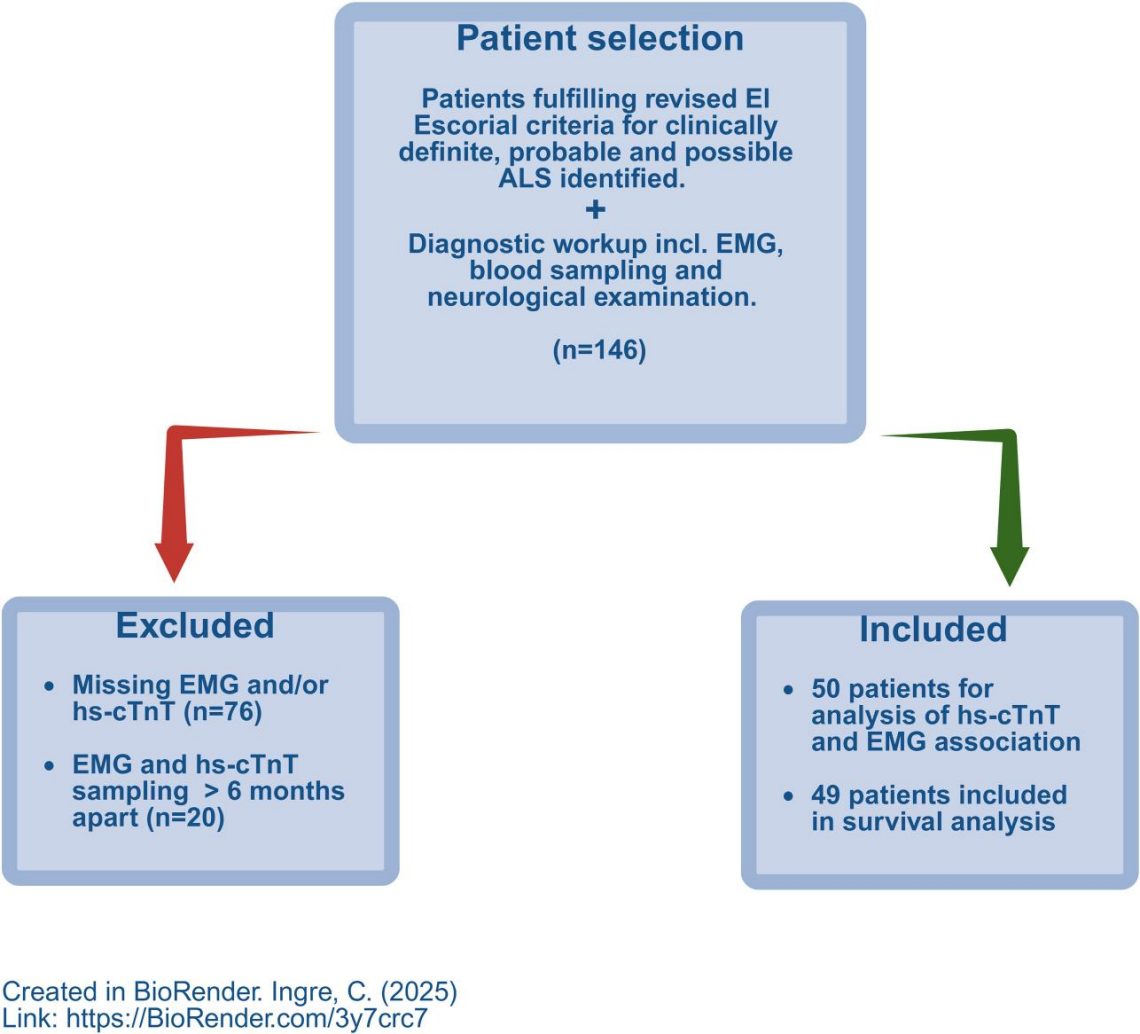
**Flow chart illustrating the process of identifying patients eligible for inclusion in the final study cohort** (https://BioRender.com/3y7crc7). Diagnostic work-up including needle EMG, plasma hs-cTnT analysed within a 6-month period from the EMG and neurological examination. EMG, Electromyography; ALS, amyotrophic lateral sclerosis; hs-cTnT, high-sensitive cardiac troponin T.

Consecutive patients who met the revised El Escorial criteria for clinically definite, probable, and possible ALS between 1 January 2014 and 31 December 2018 at the Karolinska University Hospital Neurology Clinic in Stockholm, Sweden, were screened for inclusion in the study (*n* = 146). All patients had gone through an extensive EMG investigation during their diagnostic work-up and had cTnT analysed within a six-month period from the EMG. Detailed patient history, including information on body mass index, cardiac symptoms or known cardiac co-morbidity, kidney disease, any present substernal pain, and sudden onset of dyspnoea or other cardiac-related events, was collected. Patients were categorised as having bulbar, spinal, or frontotemporal dementia (FTD) onset. Functional disability was measured using the revised ALS Functional Rating Scale.^30^ Disease progression rate was estimated by subtracting 48 (highest score of the scale) minus ALSFRS-R score obtained closest in time of the EMG examination, divided by the time in months from onset to this ALSFRS-R assessment, giving us an average monthly decline in ALSFRS-R score.^31^ We also noted King's stage as assessed by the patient's neurologist and ALSFRS-R score closest to hs-cTnT sampling. The final analysis included 50 patients for exploring the association between cTnT and disease burden as measured on EMG (Fig. 1).

The study was approved by the Swedish Ethics Review Authority (diary number 2017_1895-31_1) and followed the ethical principles of the Declaration of Helsinki. All included patients gave their written informed consent to study participation prior to their inclusion in the study.

### Sample collection and measurements

Venous blood samples were collected during the work-up of the ALS diagnosis, and in addition to cTnT, other commonly used biomarkers of cardiac and skeletal muscle injury were measured. This included CK, N-terminal pro-brain natriuretic peptide (NT-proBNP), and serum NfL (s-NfL). Patients were also tested for *SOD1* mutations and *C9orf72* repeat expansions.

All laboratory analyses of cardiac biomarkers and s-NfL were performed at Karolinska University Laboratory, a fully accredited laboratory associated with Karolinska University Hospital. For cTnT analysis, the high-sensitive cardiac troponin T (hs-cTnT) assay by Roche Diagnostics’ Elecsys fifth generation was used (Rotkreuz, Switzerland). The measurement was considered normal below 15 nanogram/L, as this is the assay´s 99th percentile value in healthy individuals and is often used as a cut-off for assessment of cardiac injury in clinical practice.^32^ The measurement of NfL was based on UmanDiagnostics’ sandwich enzyme-linked immunoassay (Umeå, Sweden; cat no 10-7001).

### Neurophysiological examination

Neurophysiological examination, including EMG, was part of the diagnostic work-up and performed at the Karolinska University Hospital Neurophysiological Clinic. We used markers of denervation activity, i.e. fibrillations and positive sharp-waves, as the main markers, examined in four body regions—cranial, cervical, thoracic, and lumbosacral, as per Awaji criteria.^21,33^ The present study included patients where the examination contained muscles from at least three of the four regions.

A muscle was considered “positive” if the EMG examination showed active fibrillations and positive sharp-waves, and a region was considered “positive” with at least one positive muscle.

The positive muscles and regions were then translated into a scoring system graded 0–4, in which 0 indicated no fibrillations/positive spike waves, while 4 represented positive findings in all four regions. The number of positive muscles was assessed in relation to the total number of muscles examined, rendering a percentage of total muscles affected/total number of muscles examined × 100 (0–100%).

### Statistical analysis

#### Descriptive statistics and correlation analysis

Descriptive analyses on the study participants’ disease characteristics were performed on the cohort level and by subgroup based on high or normal hs-cTnT level, as displayed in Table 1. The Kolmogorov-Smirnov test was used to assess the normality of the distribution of numerical variables. To assess if the difference between hs-cTnT subgroups was statistically significant for numerical variables, we used independent samples *t*-tests if data were normally distributed in both hs-cTnT subgroups using the Welch-correction, otherwise Mann–Whitney *U*-test was used. Inter-quartile range (IQR) and standard deviation (SD) were calculated for median and mean values, respectively.

**Table 1:** Patient characteristics by highly sensitive cardiac troponin T (hs-cTnT) level, with <15 ng/L considered normal

Patient factor	Elevated hs-cTnT (*n* = 27)	Normal hs-cTnT (*n* = 23)	*P*-value
Male sex, *n* (%)	17 (63)	12 (52)	0.567
Median age at diagnosis (IQR)	66 (62–73)	64 (60–70)	0.315
Spinal onset, *n* (%)	17 (63)	13 (57)^a^	0.767
Median disease duration in months at diagnosis (IQR)	14 (7–21)	11 (7–23)	0.907
Median ALSFRS-R at diagnosis (IQR)	39 (35–42)	41 (39–44)	0.061
Median s-NfL at diagnosis in ng/L (IQR)	98 (75–187)	106 (61–136)	0.397
Patients with mutation in *SOD1*, *n* (%)	0 (0)	2 (8.7)	0.214
Patients with mutation in *C9orf72*, *n* (%)	3 (11.5)	3 (13)	1.0
Mean BMI at diagnosis (SD)	24.5 (2.7)	24.5 (3.9)	0.985
Median CK in mcat/L (IQR)	3.2 (1.3–5.0)	2.2 (1.0–3.8)^b^	0.227
Median NT-proBNP in ng/L (IQR)	56 (36–94)	52 (36–102)	0.907
Median Creatinine in μmol/L (IQR)	65 (46–74)	63 (54–76)	0.311
**Median cTnT in ng/L (IQR)**	**35 (23–48)**	**10 (7–12)**	**<0.001**
Median time in days from diagnosis to hs-cTnT sampling (IQR)	62 (33–98)	51 (4–100)^c^	0.381
Median time in days from EMG to cTnT sampling (IQR)	75 (44–112)	63 (1–119)	0.370
**Median number of regions involved in EMG (IQR)**	**2 (2–3)**	**2 (1–3)**	**0.014**
**Median percentage of muscles involved in EMG (IQR)**	**50 (36–67)**	**35 (7–60)**	**0.025**
**Median King's stage at hs-cTnT sampling (IQR)**	**3 (2–3)**	**2 (1–3)**	**0.022**
Median total ALSFRS-R score at hs-cTnT sampling (IQR)	40 (33–42)	41 (38–44)	0.233
Median ALSFRS-R excl. resp. domain score at hs-cTnT sampling (IQR)	29 (24–30)	29 (26–32)	0.558
Median survival from diagnosis in days (IQR)	638 (358–1183)^d^	751 (382–1683)	0.214

IQR, inter-quartile range; ALSFRS-R, The Revised Amyotrophic Lateral Sclerosis Functional Rating Scale; s-NfL, serum neurofilament light protein; ng/L, nanogram/litre; SOD1, superoxide dismutase type 1; c9orf72, chromosome 9 open reading frame 72; BMI, body mass index; SD, standard deviation; CK, creatine kinase; mcat/L, mikrocat/litre; NT-proBNP, N-terminal prohormone of brain natriuretic peptide; µmol/L, micromole/litre; EMG, electromyography.

^a^One patient in the subgroup with normal hs-cTnT had frontotemporal dementia onset.

^b^CK had not been analysed for one patient in the group with normal hs-cTnT.

^c^One patient in the group with normal hs-cTnT had >6 months between diagnosis and hs-cTnT sampling.

^d^One patient in the group with high hs-cTnT received tracheostomy 26 days before diagnosis and was excluded from survival analysis. Statistically significant differences between patients with elevated and normal hs-cTnT are in bold.

The correlation between hs-cTnT, CK, and s-NfL, all as continuous variables, and the number of regions involved according to EMG was tested with the Kendall's rank correlation coefficient. This method was also used to assess the correlation between numerical hs-cTnT and King's stage. The correlation between hs-cTnT, CK, and s-NfL variables and the percentage of muscles affected on EMG was tested using Spearman's rank coefficient. The same method was used to examine the association between numerical hs-cTnT with total ALSFRS-R and ALSFRS-R excluding the respiratory domain scores.

#### Linear regression

First, a univariate linear regression analysis with numerical hs-cTnT as the predictor variable and percentage of muscles involved on EMG as the dependent variable was performed. We proceeded with multivariable linear regression to assess the relationship between numerical hs-cTnT and EMG disease burden while adjusting for potential confounders. In the multivariable analysis, the percentage of muscles involved was used as the dependent variable, and numerical hs-cTnT, age at diagnosis, sex, onset type, and ALSFRS-R decline rate were predictor variables. For onset type, spinal onset was coded as the reference group and since only one patient had FTD onset, bulbar and FTD onset patients were coded together as the exposed group. ALSFRS-R decline rate was noted as a positive number, indicating that a higher positive number represents a greater monthly drop in the ALSFRS-R score.

Linearity was assessed by visual examination of probability plots and scatterplots of residuals. Normality of errors was assessed with normal probability plots, and variables were transformed using the natural logarithm if not normally distributed. Equal variance of residuals was assessed with scatterplots. Multicollinearity was assessed using the variance inflation factor. Outliers were defined as ≥3 times the mean on Cook´s Distance, and the multivariable analysis was run with and without outliers. Time between EMG and hs-cTnT sampling was added as a sixth predictor in a sensitivity analysis, to assess if timing of hs-cTnT blood sampling was related to disease burden on EMG and to assess if this changed the Beta coefficients of the other predictor variables in the model (Supplementary Table 4). The overall significance of the models was assessed with the analysis of variance. How well a variable, or variables together in the adjusted models, explained the variance of the outcome was assessed with the coefficient of determination (*R*-square). Coefficients are presented as unstandardized betas with unstandardized 95% confidence intervals (CI) or standard errors (SE).

#### Survival analysis

Cox proportional hazard regression analysis was performed to assess the association between numerical hs-cTnT and survival. In the survival analysis, date of death, date of start of invasive ventilation, or 14 August 2024 (end of study) was stated as the end of follow-up. The assumption of proportional hazard was assessed visually on log minus log curves for categorical variables, including hs-cTnT high versus normal subgrouping, and no violations were found. For numerical hs-cTnT, proportional hazards and linearity assumptions were assessed by creating four equally sized (quartile) subgroups based on the hs-cTnT value. Univariate analyses were performed for age at diagnosis, onset type, high versus normal hs-cTnT, and numerical hs-cTnT, respectively. Age at diagnosis and onset type were included in addition to numerical hs-cTnT in adjusted models since the former two variables influence survival.^34^ In all analyses, *P*-values <0.05 were considered statistically significant.

## Results

**Figure 2:**
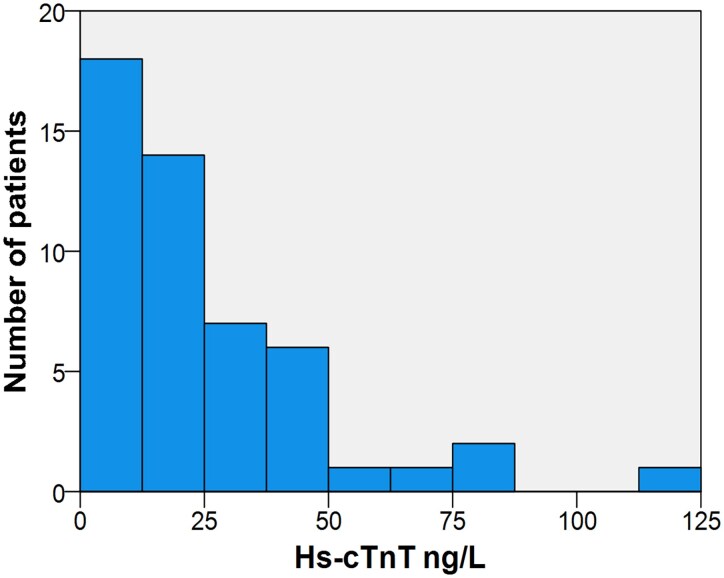
**The distribution of hs-cTnT levels in nanogram/L (*x*-axis) among study participants (*y*-axis).** Sample size (*n*) = 50. Hs-cTnT, highly sensitive cardiac troponin T.

Fifty patients diagnosed with ALS between the 1st of January 2014 and the 31st of December 2018 at the Department of Neurology at Karolinska University Hospital in Stockholm, Sweden, were enrolled in the study, of which the majority (58%, *n* = 29) were men and median age at diagnosis was 65.5 years (IQR, 61.5–70). Most of the patients (60%, *n* = 30) had spinal onset, 19 patients (38%) had bulbar onset, and one patient (2%) presented with FTD. At the time of ALS diagnosis, 14 patients had hypertension, two had atrial fibrillation, two had sleep apnoea, and one suffered from a previous heart attack. Median time from diagnosis to hs-cTnT sampling was 60.5 days (IQR, 20.3–98.5) and from EMG examination to hs-cTnT sampling was 73 days (IQR, 21–114.5). The distribution of hs-cTnT levels among study participants is shown in Fig. 2.

Patients with high levels of hs-cTnT had disease involvement in more regions and had a higher percentage of muscle involvement according to EMG, as compared to patients with normal hs-cTnT (displayed in Table 1). Disease progression as measured by King's stage was significantly higher in patients with high levels of hs-cTnT compared to patients with normal hs-cTnT. There was no significant difference in total ALSFRS-R or ALSFRS-R excluding the respiratory domain scores between high versus normal hs-cTnT patient subgroups.

**Figure 3:**
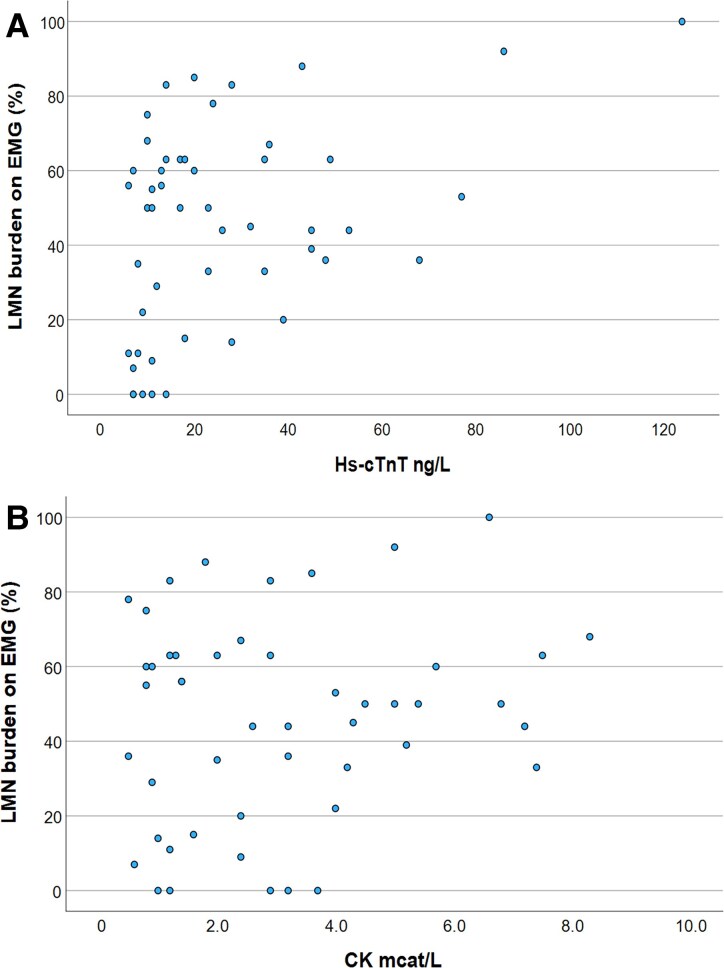
**(A–B) Scatter plots with data points representing an individual patient's LMN involvement on EMG (*y*-axis) and hs-cTnT value (*x*-axis, Fig. 3A) and CK (*x*-axis, Fig. 3B).** LMN burden on EMG in percentage on *y*-axis representing the number of positive assessed muscles (having active fibrillations and positive sharp-waves) in relation to the total number of muscles examined × 100 (0–100%). Sample size (*n*) = 50 in Fig. 3A, (*n*) = 49 in Fig. 3B. LMN, Lower motor neuron; EMG, electromyography; Hs-cTnT, highly sensitive cardiac troponin T; ng/L, nanogram/litre; CK, creatine kinase; mcat/L, mikrocat/litre.

The number of involved regions increased with hs-cTnT levels (*τ* = 0.283, *P* = 0.009) as well as the percentage of muscles affected according to EMG (*ρ* = 0.367, *P* = 0.009). There was a positive correlation between numerical hs-cTnT and King's stage (*τ* = 0.253, *P* = 0.021) but not with total ALSFRS-R or ALSFRS-R excluding the respiratory domain scores (*ρ* = −0.176, *P* = 0.220); *ρ* = −0.069, *P* = 0.632, respectively). In 49 of 50 patients, CK had been analysed from the same blood sample from which hs-cTnT was analysed, but CK correlated with neither percentage of muscles involved (*ρ* = 0.095, *P* = 0.517) nor number of regions involved according to EMG (τ = 0.159, *P* = 0.146). Scatter plots illustrating the distribution pattern of EMG involvement in relation to hs-cTnT and CK among patients are displayed in Fig. 3A and B. S-NfL had been measured from the same blood sample as hs-cTnT in all 50 patients and did not reflect the percentage of muscles involved on EMG (ρ = 0.161, *P* = 0.263), nor did they correlate with the number of regions involved (τ = 0.112, *P* = 0.293). Forty-six (92%) patients had died or received invasive ventilation by the 14th of August 2024.

### Hs-cTnT as a predictor of muscles involved in EMG

Hs-cTnT and ALSFRS-R decline rates were transformed using the natural logarithm to achieve normal distribution of their residuals. All linear regression models had a *P*-value of <0.05 for the analysis of variance. The *R*-square was 0.181 and 0.255 in the unadjusted and adjusted models, respectively.

**Figure 4:**
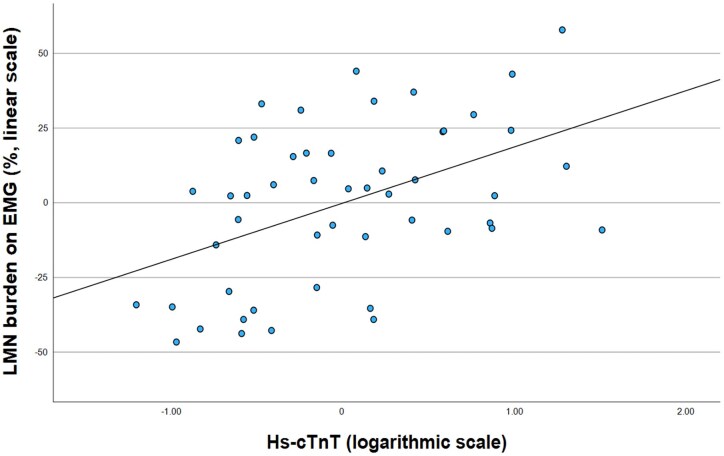
**Association between LMN involvement as detected on EMG and hs-cTnT in multivariable regression.** Partial regression plot with black line illustrating the linear relationship between LMN involvement on EMG (dependent variable, *Y*-axis) and the natural logarithm of hs-cTnT (*X*-axis) in nanogram/litre after accounting for the other independent variables in the model (age at diagnosis, sex, onset type, and ALSFRS-R decline rate). Log-transformed hs-cTnT was positively associated with LMN involvement on EMG in the adjusted linear regression ($\hat{B}$ = 18.801, *P* = 0.001). LMN burden on EMG in percentage on *y*-axis representing the number of positive assessed muscles (having active fibrillations and positive sharp-waves) in relation to the total number of muscles examined × 100 (0–100%). *F*-test of overall significance *P* < 0.05. Sample size (*n*) = 50. LMN, Lower motor neuron; EMG, electromyography; Hs-cTnT, highly sensitive cardiac troponin T.

Hs-cTnT was a significant positive predictor of disease burden as measured in percentage of muscles affected according to EMG in both univariate ($\hat{B}$ = 15.784, SE = 4.972, and *P* = 0.002) and multivariable analysis ($\hat{B}$ = 18.801, SE = 5.340, and *P* = 0.001). Age at diagnosis was a borderline significant weak predictor in the adjusted analysis ($\hat{B}$ = −0.002, SE = 0.001, *P* = 0.054), whereas onset type, sex, and ALSFRS-R decline rate were not statistically significant predictors. The results of the linear regression analyses are displayed in Supplementary Table 1 and illustrated in a partial regression plot for the association between hs-cTnT and LMN involvement on EMG in Fig. 4.

One patient in the group with normal hs-cTnT had >6 months between diagnosis and hs-cTnT sampling. In a sensitivity analysis, the exclusion of this patient did not significantly change the results of the linear regression analysis (Supplementary Table 2). Three patients were identified as outliers in the multivariable analysis with percentage of muscles affected on EMG as outcome variable, after removing these patients hs-cTnT remained a significant positive predictor ($\hat{B}$ = 20.027, SE = 5.643, *P* < 0.001) whereas age at diagnosis became a statistically significant negative predictor ($\hat{B}$ = −0.003, SE = 0.001, *P* = 0.016), illustrated in Supplementary Table 3. Timing of hs-cTnT blood sampling was not related to disease burden on EMG nor did it significantly strengthen the association between hs-cTnT, or any of the other predictors, and EMG disease burden (Supplementary Table 4).

### Association of hs-cTnT with mortality

*In the unadjusted analysis*, age at diagnosis and non-spinal onset were statistically significant predictors of survival with HR 1.000 (*P* = 0.002) and HR 3.756 (*P* = <0.001), respectively. The HR increased by 1.635 (*P* = 0.017) for every unit increase in hs-cTnT. Change from normal to high hs-cTnT group (≥15 nanogram/L) increased the HR by 1.824 (*P* = 0.060), hence not statistically significant.

*In the adjusted analysis*, when adding age at diagnosis in addition to transformed numerical hs-cTnT to the survival model, the latter was no longer a statistically significant predictor of survival (HR, 1.413; *P* = 0.105). Cox regression results are listed in Supplementary Table 5.

## Discussion and conclusions

To our knowledge, this is the first study examining the correlation between hs-cTnT and skeletal muscle affection in patients with ALS. In this study, we demonstrate a clear association between levels of hs-cTnT and muscle affection on EMG and show a correlation between hs-cTnT and both the percentage of muscles and the number of regions involved. This was not true for the established biomarkers CK and NfL.

CK has been shown to correlate with sex (higher levels in male patients), site of onset (higher levels in spinal onset), and spontaneous activity in EMG in patients with ALS,^35^ but CK was outperformed by hs-cTnT, indicating that this biomarker is more sensitive to detect LMN involvement.

In a previous study, CSF NfL levels were significantly associated with the extent of LMN degeneration, suggesting that the damaged motor neurons in the anterior horn of the spinal cord, which are rich in large axons, significantly contribute to the release of NfL in CSF.^36^ We did not find a correlation between EMG findings and NfL, suggesting again hs-cTnT as a more sensitive biomarker.

As expected, hs-cTnT correlated with King's stage but somewhat surprisingly not with total ALSFRS-R or ALSFRS-R excluding the respiratory domain scores. It is noteworthy that serum NfL has been described to negatively correlate with the ALSFRS-R score.^37^ The lack of correlation between hs-cTnT and ALSFRS-R is in contrast with a study by Castro-Gomez, S. *et al*.^29^ However, in that study, the disease duration was 3.1 and 2.5 years for patients with spinal and bulbar onset, respectively. In the present study, we used King's stage and ALSFRS-R score obtained closest to hs-cTnT sampling, which was drawn from the patient´s blood approximately two months after diagnosis (Table 1). Hence, patients in the present study were in a significantly earlier disease stage. Given that King's stage assesses the anatomical spread of ALS based on the number of affected regions, ALSFRS-R detects a functional deficit due to the disease.^38^ A sign of motor neuron damage in one region, according to King's stage, will not always, especially at earlier disease stages, lead to a functional impairment in that region, which might explain the lack of correlation between hs-cTnT and ALSFRS-R in the present study. With these aspects in mind, our interpretation of the results of the present study is that hs-cTnT can serve as a progression marker that correlates with ALS aggressiveness. Hs-cTnT was not a statistically significant predictor of survival in multivariable Cox regression, consistent with a study by Kläppe *et al*., which included 2.5 times more patients in the survival analysis than the present study.^7^

The number of patients identified with elevated hs-cTnT levels in our cohort (58%) is similar to previous publications, e.g. Castro-Gomez *et al*. and Mach *et al*., who demonstrated elevated hs-cTnT in 62.7% and 68% of ALS patients, respectively.^29,39^ The main hypothesis of the origin of hs-cTnT elevation in patients with ALS is that the cTnT is released when skeletal muscle is affected.^29,39^ Our study further explores this hypothesis, showing an association between elevated hs-cTnT levels and muscle affection according to EMG, suggesting that hs-cTnT might be a marker for levels of denervation rather than a signal of cardiac affection in ALS. This conclusion is further supported by the finding that patients with ALS with higher muscle involvement, such as spinal onset patients, show higher levels of hs-cTnT than bulbar onset ALS patients.^7^ Serum NfL did not correlate with disease burden on EMG, which might be explained by NfL representing axonal and neuronal injury rather than denervation and muscle wasting.

Further support for hs-cTnT representing skeletal muscle involvement rather than cardiac injury stems from a recent study from two independent ALS cohorts, in which serum-cTnT was found to negatively correlate with pulmonary function tests and the ALSFRS-R respiratory domain.^40^ The newly reported finding of a significant negative correlation between serum hs-cTnT concentrations and ALSFRS-R fine and motor gross functions is also consistent with this idea.^41^ In addition, studies on patients with other neuromuscular diseases suggest a non-cardiac origin of hs-cTnT measured in peripheral blood.^42-44^ Specifically, in a study by du Fay de Lavallaz *et al*. on patients with myositis and non-inflammatory myopathies, the elevated hs-cTnT levels in peripheral venous blood were thought to reflect a chronic repair mechanism in diseased skeletal muscle.^43^ This was concluded since mRNA analysis revealed an unspecific upregulation of the TNN2 gene, which codes for cTnT but not cTnI, in these patients. Furthermore, a study on patients with Pompe disease found that hs-cTnT levels were associated with skeletal muscle damage rather than acute myocardial injury.^45^

There are several strengths to this study. All blood samples for analysis of hs-cTnT were obtained at diagnosis and analysed with a validated assay in one laboratory. Given that hs-cTnT seems to increase longitudinally in ALS,^7^ and that some patients had a relatively long period between EMG examination and hs-cTnT sampling, we adjusted for time between EMG and hs-cTnT sampling in multivariable linear regression. All EMG examinations were performed, or confirmed by, a certified neurophysiologist, and all patient scores in the EMG model were assigned by the same person. We considered the likelihood of multiplicity as low, since all tests assessing the correlation between hs-cTnT and EMG disease burden pointed in the same direction (i.e. that hs-cTnT correlates positively with disease burden on EMG).

The cross-sectional design in this study has inherent limitations, with only one hs-cTnT value per patient used for prediction analysis. A longitudinal study with repeated hs-cTnT sampling might have strengthened the reliability of the present study's results. A relatively large proportion of patients were excluded due to either a lack of complete EMG data and/or hs-cTnT samples, which could introduce selection bias. That age at diagnosis was a weak negative predictor of percentage of muscles affected on EMG after, but not prior, exclusion of outliers may be due to the small sample size. Moreover, the small sample size limited the number of explanatory variables in the survival analysis to four, to prevent overfitting. This meant that we, apart from including onset type and age, which are known to predict survival, refrained from including other clinical characteristics associated with survival, such as progression or ALSFRS-R, which could have affected the results.

In addition, we acknowledge the limitations of EMG as a quantitative measure of muscle damage and lesions of the LMNs. Also, the sensitivity for detecting affected muscles with EMG would have been enhanced if at least two (i.e. one proximal and one distal) muscles were assessed per limb. Further, we did not specifically note the degree of atrophy or fasciculations, as clinical signs of LMN, but all patients had clinical signs of ALS and were eventually diagnosed as classical ALS. Moreover, we did not use validated scales for assessing LMN burden during neurological examination,^46^ and these scales should be included in future studies that aim to confirm our results. We did not control for cardiac comorbidities that could modify hs-cTnT levels in the statistical analyses. In particular, fourteen patients had hypertension, but hs-cTnT does not correlate with blood pressure.^47^ Furthermore, two patients had atrial fibrillation, and one patient had suffered from a myocardial infarction prior to ALS diagnosis, which might have influenced hs-cTnT levels, and this was not adjusted for in the analyses.

This study presents hs-cTnT, despite the current lack of standardised cut-offs for hs-cTnT in non-cardiac conditions, as a novel biomarker in neuromuscular disease. The patient population in the present study is too small to establish such a cut-off so we instead have used the accepted cardiac cut-off,^25^ which is ≥15 nanogram/L, as a threshold for categorising hs-cTnT as abnormal. Moreover, in most of the analyses in our study, including the multivariable regression with EMG disease burden as outcome, we instead used absolute (i.e. numerical) hs-cTnT to avoid using a threshold without validation to distinguish healthy from disease-affected skeletal muscles in ALS.

Additionally, we did not explore cardiac Troponin I (cTnI). In a prior study, cTnI remained normal or undetected despite elevated TnT,^44^ and for this reason, we chose not to further examine this biomarker. Degrading and regenerating skeletal muscle tissue express atypical troponin proteins (isoproteins), similar to cTnT in structure.^24^ Assays of hs-cTnT used in a laboratory cannot differentiate between hs-cTnT and its isoproteins. Hence, the detection of elevated hs-cTnT might be a false positive as previously described in myopathic disease.^28^ This might provide another explanation as to the lack of elevation of cTnI seen in prior studies despite increases in cTnT. Further, we applied the El Escorial Criteria instead of the Awaji-Shima Criteria, the latter requiring examination of four body regions. Although all patients had at least three body regions examined, there was no specific protocol for investigating the exact same muscles, which could negatively affect the validity of our results, since the probability of affected muscles showing signs of active denervation varies depending on the muscle investigated.^48^ However, we believe that it is highly unlikely that the present findings are attributable to a systematic difference between patients with high versus low hs-cTnT or high or low disease burden on EMG.

To summarise, our findings indicate that plasma hs-cTnT holds promise as a biomarker of LMN involvement in ALS. This readily available biomarker is more sensitive to LMN involvement than both CK and NfL and could potentially enhance diagnostic capabilities and serve as a complement to the diagnostic work-up. Considering our previous findings with longitudinally increasing hs-cTnT in patients with ALS, the present study indicates that hs-cTnT reflects the degree of denervation and disease spreading. Further, since hs-cTnT steadily increases throughout the disease, it could be used as a biomarker in clinical trials for ALS.

## Supplementary Material

fcaf177_Supplementary_Data

## Data Availability

The authors choose not to share the research data for privacy and ethical reasons.
