# Supplementary material for: Plasma troponin T reflects lower motor neuron involvement on electromyography in amyotrophic lateral sclerosis
Source: Brain Commun. 2025 May 6;7(3):fcaf177. doi: 10.1093/braincomms/fcaf177 (PMC12082033; doi:10.1093/braincomms/fcaf177)
Supplement: fcaf177_Supplementary_Data [file fcaf177_supplementary_data.pdf]

Supplementary material – Plasma troponin T reflects lower motor neuron involvement on electromyography in amyotrophic lateral sclerosis

	Unadjusted model with 95% CI				Adjusted model with 95% CI			
Predictor variable	Unstandardised beta	Lower	Upper	p-value	Unstandardised beta	Lower	Upper	p-value
**Plasma hs-cTnT**	**15.784**	**6.038**	**25.529**	**0.002**	**18.801**	**8.039**	**29.562**	**0.001**
Age at diagnosis	n/a				– 0.002	– 0.004	0.000	0.054
Male sex	n/a				– 2.445	– 17.056	12.166	0.738
Non-spinal onset	n/a				3.747	– 12.251	19.745	0.639
ALSFRS-R decline rate	n/a				1.231	–6.562	9.025	0.752

Supplementary Table 1. Study of the association between LMN involvement on EMG during diagnostic workup (dependent variable) and predictor variables using univariate and multivariable linear regression. Hs-cTnT was a significant positive predictor of LMN burden on EMG in both univariate ($\hat{B}$=15.784, SE=4.972, p=0.002) and multivariable analysis ($\hat{B}$ =18.801, SE=5.340, p=0.001). LMN burden on EMG in percentage representing the number of positive assessed muscles (having active fibrillations and positive sharp-waves) in relation to the total number of muscles examined x 100 (0 – 100%). Plasma hs-cTnT and ALSFRS-R decline rate were transformed using the natural logarithm prior inclusion in the regression models. Non-spinal onset represents patients with bulbar (n=19, 38%) and frontal lobe dementia (n=1, 2%) onset. ALSFRS-R decline rate defined as monthly drop in ALSFRS-R score and denoted as a positive number in the model. F-test of overall significance $p<0.05$. Sample size (n)=50. Abbreviations: CI: confidence interval; hs-cTnT: high-sensitive cardiac troponin T; ALSFRS-R: The Revised Amyotrophic Lateral Sclerosis Functional Rating Scale; n/a: not applicable; LMN: lower motor neuron; EMG: electromyography

	Adjusted model with 95% CI			
Predictor variable	Unstandardised beta	Lower	Upper	p-value
**Plasma hs-cTnT**	**19.216**	**8.268**	**30.164**	**<0.001**
Age at diagnosis	– 0.002	– 0.004	0.000	0.090
Male sex	– 1.759	– 16.686	13.168	0.813
Non-spinal onset	4.164	– 12.033	20.361	0.607
ALSFRS-R decline rate	1.239	– 6.620	9.098	0.752

Supplementary Table 2. Multivariable regression with LMN involvement on EMG as dependent variable with one patient with > 6 months between diagnosis and sampling of hs-cTnT in blood removed (n=49 in analysis). Exclusion of this patient did not significantly change the results of the regression analysis. LMN burden on EMG in percentage representing the number of positive assessed muscles (having active fibrillations and positive sharp-waves) in relation to the total number of muscles examined x 100 (0 – 100%). Non-spinal onset represents patients with bulbar (n=19, 38%) and frontal lobe dementia (n=1, 2%) onset. ALSFRS-R decline rate defined as monthly drop in ALSFRS-R score and denoted as a positive number in the model. F-test of overall significance $p<0.05$. Abbreviations: CI: confidence interval; hs-cTnT: high-sensitive cardiac troponin T; ALSFRS-R: The Revised Amyotrophic Lateral Sclerosis Functional Rating Scale; LMN: lower motor neuron; EMG: electromyography

	Adjusted model with 95% CI			
Predictor variable	Unstandardised beta	Lower	Upper	p-value
**Plasma hs-cTnT**	**20.027**	**8.631**	**31.423**	**<0.001**
**Age at diagnosis**	**– 0.003**	**– 0.005**	**–0.001**	**0.016**
Male sex	– 3.555	– 17.843	10.732	0.618
Non-spinal onset	5.973	– 10.021	21.968	0.455
ALSFRS-R decline rate	2.627	– 5.195	10.449	0.501

Supplementary Table 3. Multivariable regression with LMN involvement on EMG as dependent variable with three patients identified as outliers removed (n=47 in analysis). Hs-cTnT remained a significant positive predictor ($\hat{B}$=20.027, SE= 5.643, p<0.001) whereas age at diagnosis became a statistically significant negative predictor ($\hat{B}$=–0.003, SE=0.001, p=0.016) after removal of these three patients. LMN burden on EMG in percentage representing the number of positive assessed muscles (having active fibrillations and positive sharp-waves) in relation to the total number of muscles examined x 100 (0 – 100%). Non-spinal onset represents patients with bulbar (n=19, 38%) and frontal lobe dementia (n=1, 2%) onset. ALSFRS-R decline rate defined as monthly drop in ALSFRS-R score and denoted as a positive number in the model. F-test of overall significance p<0.05. Abbreviations: CI: confidence interval; hs-cTnT: high-sensitive cardiac troponin T; ALSFRS-R: The Revised Amyotrophic Lateral Sclerosis Functional Rating Scale; LMN: lower motor neuron; EMG: electromyography

Adjusted model with 95% CI				
Predictor variable	Unstandardised beta	Lower	Upper	p-value
**Plasma hs-cTnT**	**18.510**	**7.403**	**29.618**	**0.002**
Age at diagnosis	–0.002	–0.004	0.000	0.058
Male sex	–2.152	–17.098	12.794	0.773
Non-spinal onset	3.333	–13.154	19.819	0.686
ALSFRS-R decline rate	1.505	–6.649	9.659	0.712
Time between EMG and hs-cTnT sampling	0.530	–3.515	4.575	0.793

Supplementary Table 4. Multivariable regression with LMN involvement on EMG as dependent variable with time between EMG and hs-cTnT sampling added as a sixth predictor variable. Timing of hs-cTnT blood sampling was not related to LMN burden on EMG nor did it significantly strengthen the association between hs-cTnT, or any of the other predictors, and LMN burden on EMG. LMN burden on EMG in percentage representing the number of positive assessed muscles (having active fibrillations and positive sharp-waves) in relation to the total number of muscles examined x 100 (0 – 100%). Non-spinal onset represents patients with bulbar (n=19, 38%) and frontal lobe dementia (n=1, 2%) onset. ALSFRS-R decline rate defined as monthly drop in ALSFRS-R score and denoted as a positive number in the model. F-test of overall significance $p<0.05$. Sample size (n)=50. Abbreviations: CI: confidence interval; hs-cTnT: high-sensitive cardiac troponin T; ALSFRS-R: The Revised Amyotrophic Lateral Sclerosis Functional Rating Scale; LMN: lower motor neuron; EMG: electromyography

Model	Variable	Hazard ratio	p-value
hs-cTnT	**hs-cTnT**	**1.635**	**0.017**
Age at diagnosis	**Age at diagnosis**	**1.000**	**0.002**
Onset type	**Onset type**	**3.756**	**<0.001**
hs-cTnT + Age at diagnosis	hs-cTnT	1.413	0.105
	**Age at diagnosis**	**1.000**	**0.009**
hs-cTnT +Onset type	**hs-cTnT**	**1.948**	**0.003**
	**Onset type**	**4.470**	**<0.001**
hs-cTnT + Age at diagnosis + Onset type	**hs-cTnT**	**1.735**	**0.016**
	**Age at diagnosis**	**1.000**	**0.030**
	**Onset type**	**4.083**	**<0.001**
hs-cTnT + Age at diagnosis + Onset type + hs-cTnT * Age at diagnosis	hs-cTnT	16.319	0.197
	Age at diagnosis	1.000	0.144
	**Onset type**	**4.117**	**<0.001**
	hs-cTnT * Age at diagnosis	1.000	0.303
hs-cTnT + Age at diagnosis + Onset type + hs-cTnT * Onset type	**hs-cTnT**	**2.247**	**0.012**
	**Age at diagnosis**	**1.000**	**0.028**
	**Onset type**	**21.310**	**0.045**
	hs-cTnT * Onset type	0.594	0.277

Supplementary Table 5. Unadjusted and adjusted Cox regression results using transformed numerical hs-cTnT, age at diagnosis and onset type including interaction terms. Numerical hs-cTnT was associated with shorter survival (univariate analysis, HR=1.635, p=0.017) but not after adjusting for age at diagnosis (HR=1.413, p=0.105). For onset type spinal onset was coded as reference group and patients with bulbar (n=19, 38%) and frontal lobe dementia (n=1, 2%) coded together as exposed group. One patient received tracheostomy 26 days before ALS diagnosis and was excluded from survival analysis (n=49 in analysis). Abbreviations: CI: confidence interval; hs-cTnT: high-sensitive cardiac troponin T; ALS: amyotrophic lateral sclerosis
